# A Single Far-Field Deep Learning Adaptive Optics System Based on Four-Quadrant Discrete Phase Modulation

**DOI:** 10.3390/s20185106

**Published:** 2020-09-08

**Authors:** Xuejing Qiu, Tao Cheng, Lingxi Kong, Shuai Wang, Bing Xu

**Affiliations:** 1 Key Laboratory of Adaptive Optics, Institute of Optics and Electronics, Chinese Academy of Sciences, Chengdu 610209, Sichuan, China; qiuxuejing18@mails.ucas.ac.cn (X.Q.); chengtao@ioe.ac.cn (T.C.); konglingxi18@mails.ucas.ac.cn (L.K.); wangshuai@ioe.ac.cn (S.W.); 2 Institute of Optics and Electronics, Chinese Academy of Sciences, Chengdu 610209, Sichuan, China; 3 University of Chinese Academy of Science, Beijing 100049, China

**Keywords:** adaptive optics, convolutional neural network, four-quadrant discrete phase modulation, aberration correction, wavefront reconstruction

## Abstract

In adaptive optics (AO), multiple different incident wavefronts correspond to a same far-field intensity distribution, which leads to a many-to-one mapping. To solve this problem, a single far-field deep learning adaptive optics system based on four-quadrant discrete phase modulation (FQDPM) is proposed. Our method performs FQDPM on an incident wavefront to overcome this many-to-one mapping, then convolutional neural network (CNN) is used to directly predict the wavefront. Numerical simulations indicate that the proposed method can achieve precise high-speed wavefront correction with a single far-field intensity distribution: it takes nearly 0.6ms to complete wavefront correction while the mean root mean square (RMS) of residual wavefronts is 6.3% of that of incident wavefronts, and the Strehl ratio of the far-field intensity distribution increases by 5.7 times after correction. In addition, the experiment results show that mean RMS of residual wavefronts is 6.5% of that of incident wavefronts and it takes nearly 0.5 ms to finish wavefront reconstruction, which verifies the correctness of our proposed method.

## 1. Introduction

Adaptive optics (AO) has played an important role in beam shaping [1], super-resolution microscopy [2], human eye imaging [3], and so on. AO is composed of three key elements: a wavefront sensor (WFS) to measure distorted wavefronts, a wavefront controller that outputs control signals to a wavefront corrector based on information acquired by WFS, and a wavefront corrector such as a deformable mirror (DM) to provide wavefront correction. Conventional WFSs include Shack–Hartmann wavefront sensors [4], curvature sensors [5], plenoptic sensors [6], pyramid wavefront sensors [7], and so on. Although these WFSs have achieved real-time closed-loop wavefront detection, they have delicate optical structures, and the implementation cost is high.

Phase retrieval (PR) can directly detect wavefront phases using far-field intensity distribution. PR can work without WFS, as it has no need for calibration and implementation cost is low. Classic PR algorithms include Gerchberg–Saxton algorithm (GS) [8], phase diversity (PD) algorithm [9,10], and so on. However, PR algorithms need multiple iterations to converge, and their real-time performance is poor. In addition, PD algorithms require a camera such as a charge coupled device (CCD) to make multiple measurements on focal and defocus planes, and algorithms have limited applications in wavefront detection.

Convolutional neural network (CNN), as a branch of deep learning, has excellent performance rates in image processing. In recent years, scholars have begun to use the nonlinear fitting characteristics of CNN to acquire wavefront aberration information based on a single far-field intensity distribution image [11], achieving high wavefront detection accuracy with a simple optical system structure. However, in AO, far-field intensity distribution corresponds to multiple different incident wavefronts. This many-to-one mapping relationship means that, in supervised leaning, a sample can be related to many labels, so CNN cannot handle this kind of many-to-one mapping relationship. For this reason, scholars can only achieve precise wavefront reconstruction under small aberrations [12,13] with a single far-field intensity distribution. Authors in [14,15] applied the idea that regarding PD, far-field intensity distributions on focal and defocus planes are acquired to overcome many-to-one mapping. CCD measuring on a defocus plane is equivalent to introducing a defocus aberration, and mapping from a far-field intensity distribution to the incident wavefront is no longer a many-to-one mapping. Nevertheless, these algorithms require multiple measurements on focal and defocus planes, their system structures are complex, and the algorithms have limited applications.

In this paper we analyze the origin of the many-to-one mapping relationship and propose a single far-field CNN-AO based on four-quadrant discrete phase modulation (FQDPM). Our method overcomes many-to-one mapping by modulating the incident wavefront with FQDPM, and it reduces system computing time by replacing multiple iterations with CNN. This paper is arranged as follows. In Section 2, we introduce the working principle of FQDPM-based CNN-AO. In Section 3, numerical simulations and corresponding analyses are given. In Section 4, relevant experiments are built to verify the correctness of our theory, while conclusions follow in Section 5.

## 2. Working Principle of FQDPM-Based CNN-AO

The relationship between the complex amplitude of far-field $U_{far}\left(x_{0},y_{0}\right)$ and pupil plane $U_{near}\left(x,y\right)$ can be expressed as

(1) $$U_{far}\left(x_{0},y_{0}\right)=\iint^{\;}U_{near}\left(x,y\right)exp\left[-i2\pi \left(ux+vy\right)\right]dxdy^{\;}$$

(2) $$U_{near}\left(x,y\right)=A_{near}\left(x,y\right)exp\left[i\varphi \left(x,y\right)\right]$$

where (*x*_0_, *y*_0_) and (*x*, *y*) are rectangular coordinates on imaging and pupil planes, respectively, *u* and *v* are spatial frequencies *u* = $\frac{x_{0}}{\lambda f}$ and *v* = $\frac{y_{0}}{\lambda f}$, λ is wavelength, *f* is focal length, $A_{near}\left(x,y\right)$ represents the amplitude of the incident wavefront, and φ(x,y) is the wavefront phase. Assuming that the system is under uniform irradiation, $A_{near}\left(x,y\right)$ can be normalized to 1. By rotating wavefront φ(x,y) 180 degrees and then flipping it, we gain a new wavefront $\varphi^{\prime }\left(x,y\right)=-\varphi \left(-x,-y\right)$. According to Euler’s formula, $U_{far}\left(x_{0},y_{0}\right)$ and $U_{far}^{\prime }\left(x_{0},y_{0}\right)$ can be newly expressed as

(3) $$U_{far}\left(x_{0},y_{0}\right)=\iint^{\;}\left\{\begin{array}{c} \cos\left[\varphi \left(x,y\right)-2\pi \left(ux+vy\right)\right]\\ +i\left[\varphi \left(x,y\right)-2\pi \left(ux+vy\right)\right] \end{array}\right\}dxdy$$

(4) $$U_{far}^{\prime }\left(x_{0},y_{0}\right)=\iint^{\;}\left\{\begin{array}{c} \cos\left\{\varphi \left(-x,-y\right)-2\pi \left[u\left(-x\right)+v\left(-y\right)\right]\right\}\\ -i\sin\left\{\varphi \left(-x,-y\right)-2\pi \left[u\left(-x\right)+v\left(-y\right)\right]\right\} \end{array}\right\}d\left(-x\right)d\left(-y\right)$$

where $U_{far}\left(x_{0},y_{0}\right)$ and $U_{far}^{\prime }\left(x_{0},y_{0}\right)$ are corresponding complex amplitudes of φ(x,y) and $\varphi^{\prime }\left(x,y\right)$, respectively. According to Equations (3) and (4), $U_{far}\left(x_{0},y_{0}\right)$ and $U_{far}^{\prime }\left(x_{0},y_{0}\right)$ have the same real components and opposite imaginary components. In Fourier optics, the far-field intensity distribution is equivalent to the squared modular operation of the complex amplitude; thus, the corresponding far-field intensity distribution can be expressed as

(5) $${\left|U_{far}\left(x_{0},y_{0}\right)\right|}^{2}={\left|U_{far}^{\prime }\left(x_{0},y_{0}\right)\right|}^{2}$$

where $\varphi^{\prime }\left(x,y\right)=-\varphi \left(-x,-y\right)$, $U_{far}\left(x_{0},y_{0}\right)$, and $U_{far}^{\prime }\left(x_{0},y_{0}\right)$ correspond to the same far-field intensity distribution. In supervised learning, Equation (5) means that a sample corresponds to multiple labels, the mapping relationship is morbid, and supervised learning cannot decipher which label is true. Performing a phase modulation on the incident wavefront can fix this problem. Assuming that we perform phase modulation ϕ(x,y) on incident wavefronts φ(x,y) and $\varphi^{\prime }\left(x,y\right)$, the new complex amplitude $U_{new_{far}}\left(x_{0},y_{0}\right)$ and $U_{new_{far}}^{\prime }\left(x_{0},y_{0}\right)$ can be expressed as

(6) $$U_{new_{far}}\left(x_{0},y_{0}\right)=\iint^{\;}\left\{\begin{array}{c} \cos\left[\varphi \left(x,y\right)+\phi \left(x,y\right)-2\pi \left(ux+vy\right)\right]\\ +i\sin\left[\varphi \left(x,y\right)+\phi \left(x,y\right)-2\pi \left(ux+vy\right)\right] \end{array}\right\}dxdy$$

(7) $$U_{new_{far}}^{\prime }\left(x_{0},y_{0}\right)=\iint^{\;}\left\{\begin{array}{c} \cos\left[\varphi \left(x,y\right)-\phi \left(-x,-y\right)-2\pi \left(ux+vy\right)\right]\\ -i\sin\left[\varphi \left(x,y\right)-\phi \left(-x,-y\right)-2\pi \left(ux+vy\right)\right] \end{array}\right\}dxdy$$

According to Equations (6) and (7), when ϕ(x,y)≠−ϕ(−x,−y), $U_{new_{far}}\left(x_{0},y_{0}\right)$ and $U_{new_{far}}^{\prime }\left(x_{0},y_{0}\right)$ no longer correspond to the same far-field intensity distribution, and a pair of rotating and flipped incident wavefronts will not be the same again.

The phase modulation ϕ(x,y) must be discrete because when ϕ(x,y) is continuous, incident wavefronts φ(x,y) and $\varphi^{\prime }\left(x,y\right)=-\Phi \left(x,y\right)-\Phi \left(-x,-y\right)-\varphi \left(-x,-y\right)$ still correspond to the same far-field intensity distribution, so continuous phase modulation cannot fundamentally destroy the many-to-one mapping relationship. Discrete phase modulation is not impacted by this problem because $\varphi^{\prime }\left(x,y\right)$ does not exist when the incident wavefront is continuous.

FQDPM divides a unit circle into four quadrants: the first and third quadrants generate a phase step of $\frac{-\pi }{4}$, while the second and fourth quadrants generate a phase step of $\frac{\pi }{4}$. FQDPM is one of the simplest discrete phase modulations that meet the requirements of phase modulation. In addition, FQDPM aberration plates are easy to manufacture. For these reasons, FQDPM is the chosen modulation. Assuming that the expression of FQDPM is δ(x,y), δ(x,y) can be expressed as

(8) $$\delta \left(x,y\right)=\{\begin{array}{c} \frac{-\pi }{4}xy\geq 0\\ \frac{\pi }{4}xy<0 \end{array}$$

**Figure 1:**
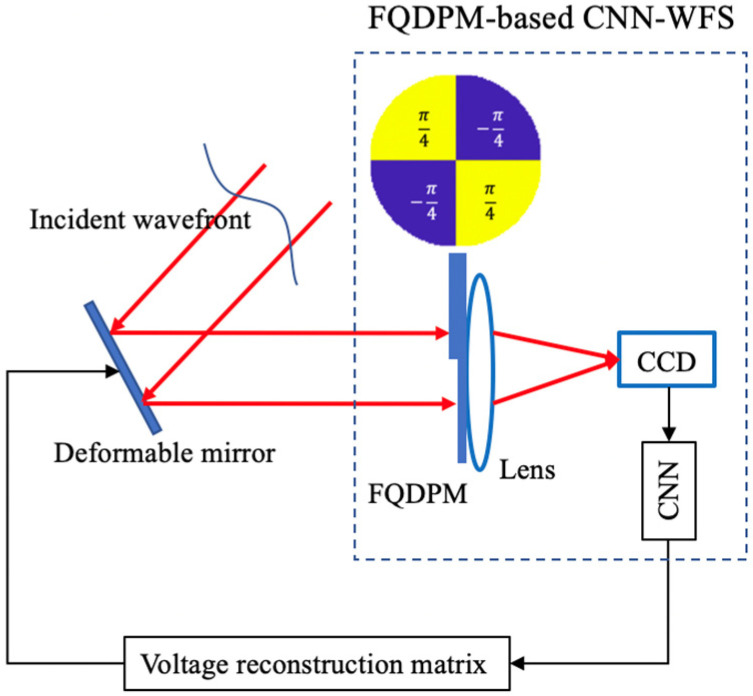
Schematic diagram of the four-quadrant discrete phase modulation (FQDPM)-based convolutional neural network adaptive optics (CNN-AO) system.

When we perform FQDPM on an incident wavefront, according to Equations (6)–(8), FQDPM can break the many-to-one mapping that multiple incident wavefronts correspond to in a same far-field intensity distribution. Due to this, it is feasible to use CNN to fit the mapping from the far-field intensity distribution to the wavefront aberration information. A FQDPM-based CNN-AO system is shown in Figure 1, and a FQDPM aberration plate is added to the optical system between a DM and a positive lens. A FQDPM-based CNN-AO system is composed of three key elements: a FQDPM-based CNN-WFS to measure the far-field intensity distribution on a lens focal plane and calculate wavefront aberration information, a voltage reconstruction matrix that outputs voltage control signals to DM based on calculated aberration information, and a DM to provide wavefront correction on the basis of input voltage control signals.

**Figure 2:**
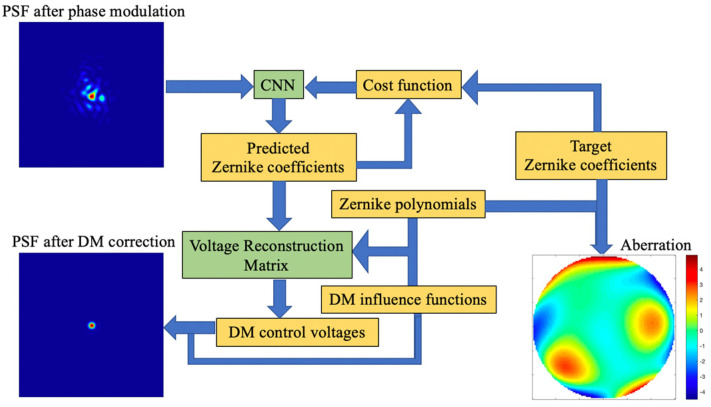
Working principle of the FQDPM-based CNN-AO.

The working principle of FQDPM-based CNN-AO is shown in Figure 2. CNN is used to fit the nonlinear mapping between modulated PSFs and Zernike coefficients. Errors between Zernike coefficients are predicted by CNN, and target coefficients are sent to the cost function, while CNN adjusts its parameters based on gradient information of the cost function; this process is called CNN training. After CNN training, CNN directly outputs Zernike coefficients according to input modulated PSFs, then the voltage reconstruction matrix converts predicted coefficients acquired by CNN into DM control voltages. DM generates corresponding surface shapes to compensate for the incident wavefront on the basis of control voltages; thus, the system achieves closed-loop correction. Numbers of CCD pixels used in FQDPM-based CNN-WFS are 120 × 120 pixels.

## 3. Numerical Simulations

**Figure 3:**
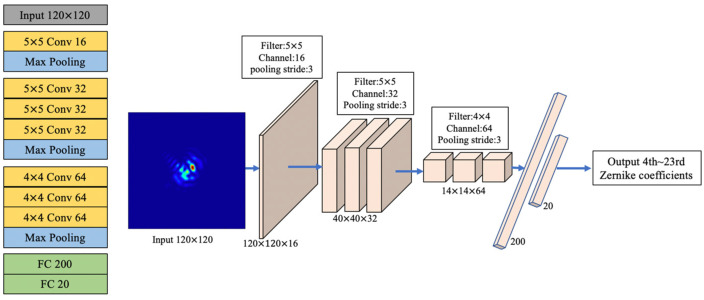
CNN architecture.

Relevant parameters of the numerical simulations are as follows: wavelength was 1064 nm, radius of the optical aperture was 8 mm, focal length of the lens was 1000 mm, CCD pixel size was 10 μm×10 μm, and number of pixels in CCD was 120 ×120 pixels. CNN architecture and its hyper parameters are shown in Figure 3. It has 13 layers, including 7 convolutional layers, 3 pooling layers, and 2 fully connected (FC) layers. A PSF image with a size of 120 ×120 pixels was processed in the input layer; the number of channels of each convolution layer was 16, 32, 32, 32, 64, 64, and 64, respectively; the size of each convolution kernel was 5 × 5, 5 × 5, 5 × 5, 5 × 5, 4 × 4, 4 × 4, and 4 × 4, respectively; the pooling layer used max pooling with a stride of 3; and the number of neurons in FC layers were 200 and 20, respectively. CNN selected ReLU as the activation function and was applied in all convolutional layers and the first FC layer. This task is a regression task, so we chose the mean square error (MSE) function as CNN loss function. During CNN training, the batch size was set to 100, the epoch was set to 200, and the Adam function with an initial learning rate 10^−3^ was chosen as the CNN gradient descent function. Workstation configurations were Intel Core i7 9700 K 3.6 GHz, Kingston 64 GB, and NVIDIA GeForce RTX2080Ti. Training and testing were finished on the GPU. It took nearly 14 min to complete CNN training and 0.6 ms for our method to complete one round of wavefront correction.

### 3.1. Generate Dataset

A series of 10,000 incident wavefronts were randomly generated based on the 4th to 23rd Zernike coefficients. After modulation by FQDPM, new incident wavefronts were measured by CCD to obtain PSFs. The range of each Zernike coefficients was within ±0.5 μm. Samples in training and test sets were independently and identically distributed, so we randomly selected 9000 PSFs and corresponding Zernike coefficients as samples and labels in the training set, respectively. The remaining 1000 PSFs and corresponding Zernike coefficients were used as samples and labels in the test set, respectively. Labels were 20 × 1 column vectors. A training set was used so CNN could learn the mapping relationship between the modulated PSFs and corresponding Zernike coefficients. A test set was used to evaluate the accuracy of our method.

### 3.2. Results and Analyses of Simulations

**Figure 4:**
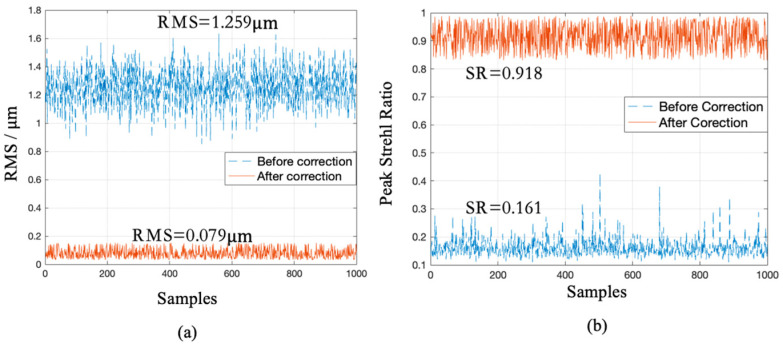
RMS (**a**) and SR (**b**) before and after correction of 1000 samples in the test set.

In order to evaluate the correction results of numerical simulations, the root mean square (RMS) of residual wavefronts and Strehl ratio (SR) of the far-field intensity distribution were selected as evaluation indexes. Figure 4a,b shows RMS and SR, respectively, before and after correction corresponding to 1000 samples in the test set. It is known from Figure 4a that the mean RMS of incident wavefronts before correction was 1.259 μm, and mean RMS of residual wavefronts after correction dropped to 0.079 μm; thus, the mean RMS of the corrected residual wavefronts was 6.3% of that of incident wavefronts. In Figure 4b, the mean SR before correction was 0.161, and the mean SR after correction rose to 0.918; thus, the mean SR increased by 5.7 times after correction. Results in Figure 4 indicated that the well-trained CNN combined with the voltage reconstruction matrix could effectively and directly output DM control voltages to make AO closed-loop based on a single modulated PSF. Our method converged the algorithm after one calculation, ensuring that no more iterations were needed to optimize the performance index, which led to a greatly reduced computing time.

**Figure 5:**
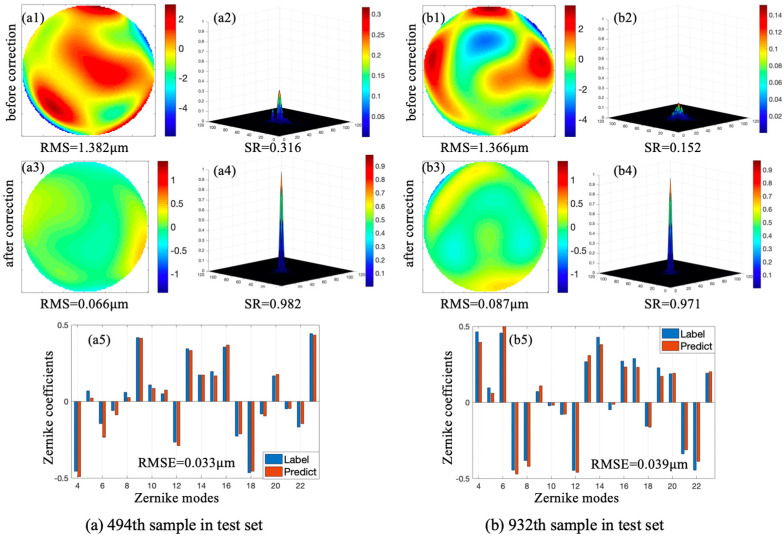
Correction results of 494th (**a**) and 932nd (**b**) samples in test set.

Two samples were randomly selected from the test set. Figure 5 exhibits two corresponding incident wavefronts, initial PSFs, residual wavefronts, and PSFs after correction. Fitting results between predicted Zernike coefficients and labels are also shown in Figure 5; the scales on the *X*-axis and *Y*-axis represent Zernike modes and Zernike coefficients, respectively. In each Zernike mode, the left pillars represent the labeled Zernike coefficients while the right pillars represent Zernike coefficients predicted by CNN. Figure 5a,b corresponds to the 494th and 932nd samples in the test set, respectively. Root-mean-square error (RMSE) was introduced to evaluate fitting results of Zernike coefficients predicted by the proposed method.

In Figure 5a, the initial RMS and SR corresponding to the 494th sample in the test set were 1.382 μm and 0.316 respectively. After correction, RMS and SR were 0.066 μm and 0.982, respectively, and RMSE between predicted Zernike coefficients and labels was 0.033 μm. In Figure 5b, the initial RMS and SR corresponding to the 932nd sample in the test set were 1.366 μm and 0.152, respectively. After correction, RMS and SR were 0.087 μm and 0.971, respectively, and RMSE between predicted Zernike coefficients and labels was 0.039 μm. CNN achieved good fitting and the method achieved precise aberration correction.

## 4. Experiments

### 4.1. Experimental Setup

**Figure 6:**
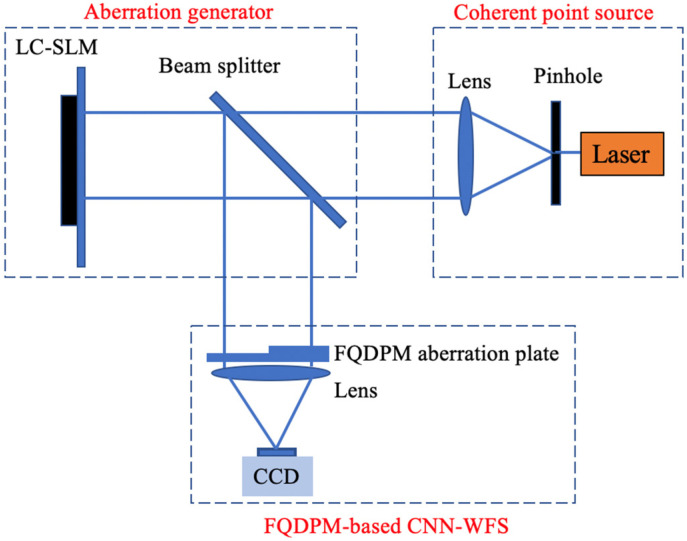
Schematic diagram of the optical system structure.

It is known from Figure 2 in Section 2 that the voltage reconstruction matrix needs exact expressions of DM influence functions. In simulations, DM influence functions can be precisely defined, but in experiments they should be measured by a WFS such as Shack–Hartmann wavefront sensor or interferometer. To simplify procedures of experiments, in this section we built a wavefront detection system based on a liquid crystal spatial light modulator (LC-SLM) and ignored the wavefront correction. A schematic diagram of the optical system structure is shown in Figure 6. Our system contained three parts: a coherent point source, an aberration generator, and a FQDPM-based CNN-WFS. The coherent source was composed of a laser, a pinhole, and a positive lens. The pinhole was placed at the focal point of the positive lens to generate a collimated beam. The aberration generator was composed of a beam splitter and LC-SLM. When the incident beam is reflected by LC-SLM, LC-SLM adds additional aberrations to the incident beam to change its wavefront phase distribution. The FQDPM-based CNN-WFS contained a FQDPM aberration plate, a positive lens, and a CCD which was placed at the focal plane of the lens. A distorted beam was reflected by a beam splitter and reached FQDPM-based CNN-WFS, the FQDPM aberration plate then performed FQDPM on the incident beam, and the positive lens focused the beam, and CCD measured its corresponding far-field intensity distribution.

**Figure 7:**
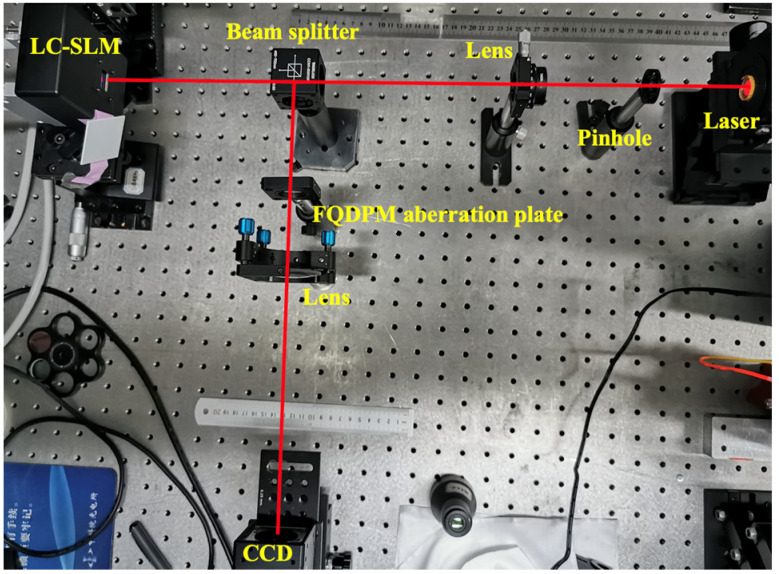
Schematic diagram of a real optical path.

A schematic diagram of the real optical path is shown in Figure 7, and relevant experimental parameters are given as follows: wavelength was 1064 μm, pinhole diameter was 10 μm, focal lengths of the lens in coherent point source and FQDPM-based CNN-WFS were 100 mm and 350 mm, respectively, CCD pixel size was 7.4 μm × 7.4 μm, and numbers of CCD pixels used in FQDPM-based CNN-WFS was 120 × 120 pixels.

According to Section 3.1, 10,000 series of samples were randomly generated according to the 4th to 23rd Zernike coefficients by LC-SLM. The range of each Zernike coefficients was within ±0.5 μm. A total of 9000 PSFs and corresponding Zernike coefficients were randomly selected as samples and labels in the training set, respectively, and the remaining 1000 PSFs and corresponding Zernike coefficients were used as samples and labels in the test set, respectively. Workstation and CNN setups were the same as mentioned in Section 3. It took nearly 14 min to complete CNN training and nearly 0.5ms for our method to reconstruct incident wavefront a single time.

### 4.2. Results and Analyses of Experiments

**Figure 8:**
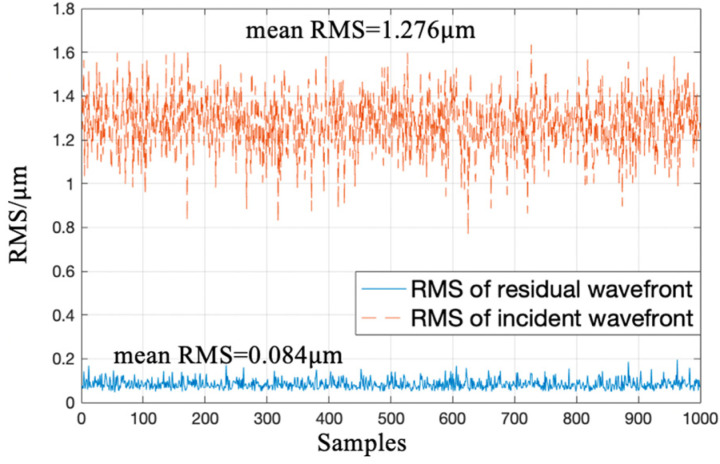
Wavefront reconstruction accuracy of samples in the test set.

Figure 8 shows RMS of residual wavefronts and RMS of incident wavefronts corresponding to 1000 samples in the test set. It is known from Figure 8 that the mean RMS of incident wavefronts was 1.276 μm, mean RMS of residual wavefronts dropped to 0.084 μm, and mean RMS of reconstructed residual wavefronts was 6.5% of that of incident wavefronts. The well-trained CNN could directly output wavefront aberration information after one calculation with a single modulated PSF.

**Figure 9:**
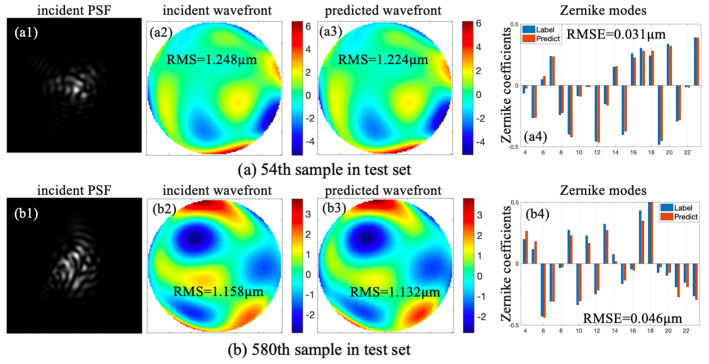
Reconstruction results of 54th (**a**) and 580th (**b**) samples in the test set.

Two samples were randomly selected from the test set. Figure 9 exhibits two incident PSFs, incident wavefronts, and predicted wavefronts. Fitting results between predicted Zernike coefficients and labels are also shown in Figure 9; the scales on the *X*-axis and *Y*-axis represent Zernike modes and Zernike coefficients, respectively. In each Zernike mode, the left pillars represent labeled Zernike coefficients and the right pillars represent Zernike coefficients predicted by CNN. Figure 9a,b corresponds to the 54th and 580th samples in the test set, respectively.

In Figure 9a, RMS of incident wavefront corresponding to the 54th sample was 1.248 μm, RMS of the predicted wavefront was 1.224 μm, and RMSE between labels and predicted coefficients was 0.031 μm. In Figure 9b, the initial RMS corresponding to the 580th sample was 1.158 μm, RMS of predicted wavefront was 1.132 μm, and RMSE between labels and predicted coefficients was 0.046 μm. According to Figure 9, surface shapes of predicted wavefronts and incident wavefronts were nearly identical, and FQDPM-based CNN-WFS could reconstruct wavefronts quickly and perfectly with a single modulated PSF.

## 5. Conclusions

In this paper we propose a single far-field FQDPM-based CNN-AO system. Our method applies FQDPM to incident wavefronts to overcome the ill-conditioned many-to-one mapping that multiple incident wavefronts correspond to a same far-field intensity distribution. We also establish a new mapping relationship between single modulated PSF and DM control voltages. Numerical simulations indicated that the mean RMS of residual wavefronts corresponding to 1000 samples in the test set dropped from 1.259 μm to 0.079 μm after correction by our proposed method, the mean SR rose from 0.161 to 0.9176, and the computing time was 0.6 ms for one correction. In the experiments we built a FQDPM-based CNN-WFS. Results showed that our proposed method achieved precise and high-speed wavefront reconstruction, the mean RMS of residual wavefronts corresponding to 1000 samples in the test set dropped from 1.276 μm to 0.084 μm, and it took nearly 0.5 ms to finish a single wavefront reconstruction.
